# The Downregulation of *LSAMP* Expression Promotes Lung Cancer Progression and Is Associated with Poor Survival Prognosis

**DOI:** 10.3390/jpm11060578

**Published:** 2021-06-20

**Authors:** Chao-Yuan Chang, Kuan-Li Wu, Yung-Yun Chang, Yu-Wei Liu, Yung-Chi Huang, Shu-Fang Jian, Yi-Shiuan Lin, Pei-Hsun Tsai, Jen-Yu Hung, Ying-Ming Tsai, Ya-Ling Hsu

**Affiliations:** 1 Graduate Institute of Medicine, College of Medicine, Kaohsiung Medical University, Kaohsiung 807, Taiwan; chaoyuah@kmu.edu.tw (C.-Y.C.); 980448kmuh@gmail.com (K.-L.W.); beryl1992@gmail.com (Y.-C.H.); chienfang1216@gmail.com (S.-F.J.); ysirenelin@gmail.com (Y.-S.L.); kanginbobo@gmail.com (P.-H.T.); jyhung@kmu.edu.tw (J.-Y.H.); yainghsu@kmu.edu.tw (Y.-L.H.); 2 Department of Anatomy, Kaohsiung Medical University, Kaohsiung 807, Taiwan; 3 Division of Pulmonary and Critical Care Medicine, Department of Internal Medicine, Kaohsiung Medical University Hospital, Kaohsiung Medical University, Kaohsiung 807, Taiwan; cyy807@gmail.com; 4 School of Medicine, College of Medicine, Kaohsiung Medical University, Kaohsiung 807, Taiwan; 5 Division of General Medicine, Department of Internal Medicine, Kaohsiung Medical University Hospital, Kaohsiung Medical University, Kaohsiung 807, Taiwan; 6 Division of Thoracic Surgery, Department of Surgery, Kaohsiung Medical University Hospital, Kaohsiung Medical University, Kaohsiung 807, Taiwan; Nipma6714@gmail.com; 7 Drug Development and Value Creation Research Center, Kaohsiung Medical University, Kaohsiung 807, Taiwan

**Keywords:** EMT, lung adenocarcinoma, LSAMP

## Abstract

Lung cancer has been a leading cause of cancer-related death for decades and therapeutic strategies for non-driver mutation lung cancer are still lacking. A novel approach for this type of lung cancer is an emergent requirement. Here we find that loss of LSAMP (Limbic System Associated Membrane Protein), compared to other IgLON family of proteins NTM (Neurotrimin) and OPCML (OPioid-binding Cell adhesion MoLecule), exhibits the strongest prognostic and therapeutic significance in predicting lung adenocarcinoma (LUAD) progression. Lower expression of *LSAMP* and *NTM*, but not *OPCML*, were found in tumor parts compared with normal parts in six LUAD patients, and this was validated by public datasets, Oncomine^®^ and TCGA. The lower expression of *LSAMP*, but not *NTM*, was correlated to shorter overall survival. Two epigenetic regulations, including hypermethylation and miR-143-3p upregulation but not copy number variation, were associated with downregulation of *LSAMP* in LUAD patients. Pathway network analysis showed that NEGR1 (Neuronal Growth Regulator 1) was involved in the regulatory loop of LSAMP. The biologic functions by *LSMAP* knockdown in lung cancer cells revealed *LSMAP* was linked to cancer cell migration via epithelial-mesenchymal transition (EMT) but not proliferation nor stemness of LUAD. Our result showed for the first time that *LSAMP* acts as a potential tumor suppressor in regulating lung cancer. A further deep investigation into the role of *LSAMP* in lung cancer tumorigenesis would provide therapeutic hope for such affected patients.

## 1. Introduction

Lung cancer is still the most deadly cancer worldwide [1,2]. In Taiwan, lung cancer has been responsible for the leading cause of cancer-related death for decades [3]. The most common causes that lead to lung cancer are tobacco smoking, air pollution either indoor or outdoor, asbestos, etc. [4]. Furthermore, LUAD harbors distinctive features of gene alterations such as epidermal growth factor receptor (*EGFR*), anaplastic lymphoma kinase-echinoderm microtubule-associated protein-like4 (*ALK-EML4*), c-ros oncogene 1 (*ROS1*), REarranged during Transfection (*RET*), and neurotrophic tropomyosin-related kinases (*NTRK*) [5]. Nowadays, the existence of these driver mutations provides therapeutic benefits via specific tyrosine kinase inhibitors (TKIs) with the greatest efforts focusing on them. However, the critical issue lies in that the LUAD patients of indistinctive gene alternations only benefit from platinum doublets and/or immunotherapy with a demise hope [6]. To overcome this difficulty, the novel mechanism and relevant treatment strategy are still necessary to pursue higher therapeutic rates.

Avoidance of metastasis is a hot target for lung cancer research. Tumors of metastatic entities contribute to more than 90% of cancer-related deaths and the aggressiveness of lung cancer enables them to metastasize by enhancing mobility, invasion, and resistance to apoptotic stimuli [7] and regains their stem-like trait [8]. The epithelial-mesenchymal transition (EMT) and mesenchymal-epithelial transition (MET) mechanisms confer tumor aggressiveness and metastasis with EMT is associated with loss of cell-cell adhesion and the gain of invasiveness [9]. At the same time, the MET causes morphologic and functional changes and the acquisition of epithelial characters to facilitate metastasis. In addition, EMT tightly contributes to the cancer stem cell (CSC) theory, which implies that only a few subpopulations of “stem-like” cells can lead to metastatic disease [9]. CSCs are cells with the ability to self-renew and generate a heterogeneous population within the tumor. The roles of EMT/MET and CSC are keys to elucidate the mechanisms of cancer metastasis.

IgLONs are an immunoglobulin (Ig) subfamily, including opioid binding protein/cell adhesion molecule–like (OPCML/OBCAM) [10], limbic system–associated membrane protein (LSAMP/LAMP) [10], neurotrimin (NTM) [11], neuronal growth regulator 1 (NEGR1/Kilon) [12], and IgLON family member 5 (IgLON5) [13]. The IgLON family consists of a group of cell adhesion molecules to regulate neurite outgrowth [14], dendritic arborization [15], and synapse formation [16]. They execute their functions by forming homophilic and heterophilic complexes along the cell surface. The functional specificity and divergence of the IgLONs are to control neuronal development and psychiatric disorders such as schizophrenia and major depression [17,18]. The behavioral changes and structural brain endophenotypes related to IgLONs are not limited to humans but also affect other mammals such as mice [18]. IgLON neural adhesion molecules not only regulate neuronal events, but also function as tumor-suppressor genes in a number of non-neural organs and tissue types [19]. IgLONs have been linked to sporadic ovarian tumors [20], gastric cancer [21], and osteosarcoma [22]; however, the IgLONs have not been well investigated in lung cancer, and elucidation of this family might be of great help in curing this disease.

To overcome the demise of hope for non-driver mutation, the advancement in genome-wide profiling technology has brought about a substantial improvement of the understanding of the genetic landscape and pathways leading to lung cancer [23,24]. Global analysis of gene expression by transcriptomic sequencing has generated a complete set of RNA transcripts (messenger RNA, micro RNA, etc.) of the genome of lung cancer in-depth [25,26]. In this study, we took advantage of the powerful next-generation sequencing (NGS) to investigate the expression of the three members of the IgLON family from six-paired adjacent normal versus tumor lung tissue samples from patients with lung cancer. This study analyzed the three members of the IgLON family—OPCML, LSAMP, and NTM—which revealed decreased LSAMP expression in patient samples and public databases, such as The Cancer Genome Atlas (TCGA) and Oncomine datasets. Furthermore, the lower the *LSAMP* was, the more aggressive lung cancer behaved. The low level of *LSAMP* conferred a shorter survival time and under the epigenetic regulation by miR-141-3p and DNA methylation. These results provided evidence that *LSAMP* regulates tumorigenesis and brings treatment hope for lung cancer. The advanced exploration of *LSAMP* in tumorigenesis might offer therapeutic hope in lung cancer.

## 2. Materials and Methods

### 2.1. Cell Lines and Cell Culture

Human lung adenocarcinoma cell lines A549 cells were purchased from the American Type Culture Collection (ATCC, Manassas, VA, USA). A549 cells were cultured in F-12K Medium (ATCC) with the supplement of 10% fetal bovine serum (FBS), 100 U/mL penicillin and 100 µg/mL streptomycin (Thermo Fisher Scientific, Boston, MA, USA). A549 was authenticated by the short tandem repeat analysis (Promega, Madison, WI, USA) and ascertained negative for mycoplasma contamination by MycoAlert™ mycoplasma detection kit (Lonza, Switzerland) every 3 months.

### 2.2. Next-Generation Sequencing

Six pairs of adjacent lung non-tumor and tumor tissues were acquired from the Division of Thoracic Surgery and Division of Pulmonary and Critical Care Medicine, Kaohsiung Medical University Hospital (Kaohsiung, Taiwan). The study protocol was reviewed and approved by the Institutional Review Board of Kaohsiung Medical University Hospital (KMUH-IRB-20130054, 24 May 2013). The freshly isolated and unfixed surgical tissues were immediately extracted for total RNA preparation. The mRNA profiles were further examined utilizing NGS using the Illumina platform (Welgene Biotechnology Company, Taipei, Taiwan) [27]. RNA and small RNA library construction were carried out using an Illumina sample preparation kit, following the protocol of the TruSeq RNA or Small RNA Sample Preparation Guide. The criteria for differentially expressed mRNA by NGS analysis were fold change >2 and fragments per kilobase million (FPKM) >0.3. Furthermore, the IgLON family, OPCML, LSAMP, and NTM were extracted for analysis.

### 2.3. Bioinformatics

The mRNA expression of IgLONs was derived from the TCGA of UALCAN (accessed on 1 January 2021; ualcan.path.uab.edu) [28]. According to the data from this website, the expression levels of IgLONs were reclassified under the criteria of lymph node metastasis (N0-N3) and tumor stage (pathologic stages 1 to 4) based on AJCC (American Joint Committee on Cancer) 8th edition. The ratio of mRNA expression in lung cancer and normal specimens (cancer vs. normal) was derived from Oncomine database (accessed on 1 January 2021; http://www.oncomine.org), Compendia biosciences, Ann Arbor, MI, USA). The criteria for analysis were >2 fold change and *p*-value < 0.0001 through the independent *t*-test. The protein expression of IgLONs was retrieved from the CPTAC of UALCAN (accessed on January 2021; ualcan.path.uab.edu) [28]. The expression levels of LSAMP, NTM but lacking OPCML, were reclassified under the criteria of lymph node metastasis (N0–N3) and tumor stage (stage 1 to 4). The relationship between IgLONs and overall survival rates in lung adenocarcinoma was assessed by either the RNA-seq or the RNA gene chip cohort in the KM plotter (accessed on 1 August 2020; http://kmplot.com/analysis/). Patients were divided into 2 groups (high and low expression) based on the best cut-off in RNA-seq cohort and by the median value in the RNA gene chip cohort. The probability of survival between the two groups was computed. The hazard ratios (95% confidence intervals) were calculated using the Cox proportional model.

### 2.4. DNA Methylation and Copy Number

The extent of *LSAMP* DNA methylation was compared according to the tissue source, the tumor stages, and the stages of lymph node metastasis on the UALCAN website (accessed on 1 January 2021; ualcan.path.uab.edu). The expression profiles of gene-specific DNA methylation data, copy number (gene level) and clinical information of patients with LUAD were extracted from the dataset TCGA Pan-Cancer (PANCAN) from the UCSC Xena website. Pearson’s correlation between *LSAMP* mRNA expression level and the copy number and/or DNA methylation was calculated using the metadata.

### 2.5. miRNAs and LSAMP Correlation

Pearson’s correlation between *LSAMP* and all microRNAs in the TCGA LUAD dataset was calculated. The miRNAs were ranked by r value and validated by TargetScan (accessed on 1 August 2020; http://www.targetscan.org/vert_72/) from the top-ranked miRNAs. Excluding the miRNAs with context++ score percentile <90, only miR-141-3p remained with a good context++ score 98th percentile.

### 2.6. Gene and Protein Interaction of LSAMP

Genes interacting with *LSAMP* are predicted by the Pathway Common website (accessed on 1 January 2021; https://www.pathwaycommons.org/; Version 12), whereas the proteins that interact with LSAMP are predicted by String Website (accessed on 1 January 2021; https://string-db.org/; Version 11.0). 

### 2.7. LSAMP Knockdown

Knockdown of *LSAMP* in A549 cells was performed using an shRNA expression system obtained from the National RNAi Core Facility (Taipei, Taiwan). The knockdown efficacy of LSAMP shRNA plasmid was determined by Immunoblot.

### 2.8. Cell Proliferation, Transwell Migration, Wound Healing, and Tumor Spheroid Formation

Cell proliferation was assessed by BrdU (Bromodeoxyuridine/5-bromo-2′-deoxyuridine) incorporation analysis for 72 h (EMD Millipore, Burlington, MA, USA), according to the manufacturer’s protocol. For migration, cells were seeded into inserts with polyester membranes with a pore size of 8 μm (EMD Millipore, Burlington, MA, USA). Complete cell culture medium was then added to the bottom wells for 48 h as a chemo-attractant. Migratory cells were visualized using crystal violet staining. Alternatively, cells were placed into a 12 well-plate at 100% confluence, and cell movement was measured by determining the migration of cells into the acellular area formatted by a sterile tip. The quantitative results of transwell migration and spheroid formation were performed using the counting method. For tumor spheroid formation, cells were seeded in ultralow-attachment plates (Corning Life Sciences, Tewksbury, MA, USA) for 7 days and the tumor spheres were assessed using an ImageXpress Micro system (Molecular Devices LLC, Sunnyvale, CA, USA).

### 2.9. Immunoblot

Cellular total protein was extracted using RIPA lysis buffer (EMD Millipore, Billerica, MA, USA) supplemented with a protease inhibitor cocktail (Sigma-Aldrich, St. Louis, MO, USA). An equal amount of cellular protein was denatured by heating, and then separated by SDS-PAGE. Proteins were transferred to PVDF membranes (EMD Millipore, Burlington, MA, USA) and probed with various primary antibodies for 4–16 h, followed by incubation with horseradish peroxidase (HRP)-conjugated secondary antibodies (Cell-Signaling Technology, Danvers, MA, USA). Signals of specific proteins were detected using a chemiluminescence kit (EMD Millipore, Burlington, MA, USA). The primary antibodies used included LSAMP (PA5-69373, PA5-51223 for IHC, ThermoFisher, Waltham, MA, USA), those not characteristic of cancer stem cells, c-Myc (Catalog # 18583, Cell-Signaling Technology, Danvers, MA, USA), OCT4 (Catalog#2890, Cell-Signaling Technology, Danvers, MA, USA), Nanog (Catalog#4903, Cell-Signaling Technology, Danvers, MA, USA), SOX2 (Catalog#3579, Cell-Signaling Technology, Danvers, MA, USA), and GAPDH (Catalog#5174, Cell-Signaling Technology, Danvers, MA, USA) and EMT markers, N-cadherin (Catalog #610920, BD Biosciences (San Jose, CA, USA)), E-cadherin (Catalog #610404, BD Biosciences, Franklin Lakes, NJ, USA), Vimentin (Catalog#550513, BD Biosciences, Franklin Lakes, NJ, USA), α-SMA (Catalog #A5228, Sigma-Aldrich, St. Louis, MO, USA), Snail (Catalog t# 3879, Cell-Signaling Technology, Danvers, MA, USA), and Slug (Catalog # 9585, Cell-Signaling Technology, Danvers, MA, USA).

### 2.10. Statistical Analysis

Results of the cell experiments are presented as mean ± standard deviation (SD). Differences between the two tested groups were compared using the Student’s *t*-test with GraphPad Prism software (7.04 version, Graphpad Software, San Diego, CA, USA). Statistical significance was defined as *p*-value < 0.05.

## 3. Results

### 3.1. The Downregulated Expressions of LSAMP and NTM Genes in Lung Adenocarcinoma

**Figure 1:**
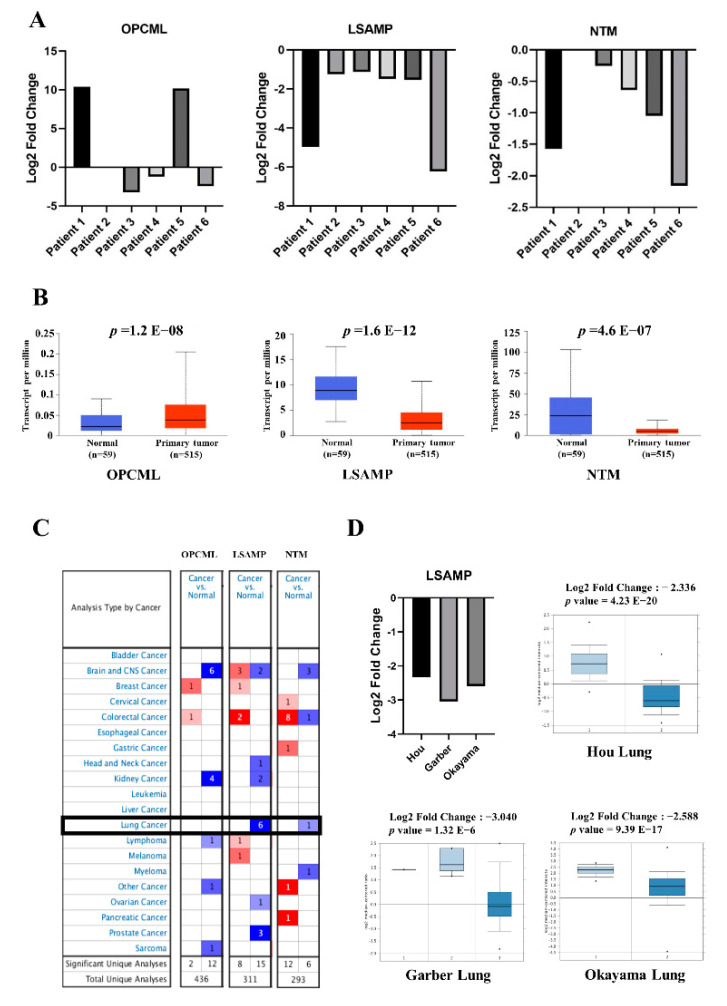
*LSAMP* mediated lung cancer tumorigenesis disclosed by transcriptomic data and bioinformatic analysis: The specimens from six human subjects were collected for IgLON family analysis. The expression levels of *OPCML*, *LSAMP* and *NTM* of six patients with LUAD (**A**). The comparative expression levels of *OPCML*, *LSAMP*, and *NTM* in tumor versus normal tissue samples were retrieved from TCGA database (**B**). The expression of *OPCML*, *LSAMP*, and *NTM* from datasets of the Oncomine was summarized (**C**), and the fold change of *LSAMP* in three different cohorts, specifically on adenocarcinoma, was extracted from the Oncomine database (**D**).

To identify the family of IgLON genes including *OPCML*, *LSAMP*, and *NTM* that regulate lung tumorigenesis, six paired normal and adenocarcinoma lung tissues were collected for analysis (Table 1). The expression levels of *LSAMP* and *NTM* in tumor tissues were lower than that in normal tissue samples of lung adenocarcinoma (LUAD) (middle and right panel, Figure 1A); however, the levels of *OPCML* were relatively inconsistent (left panel, Figure 1A). To further validate the gene expression of *OPCML*, *LSAMP*, and *NTM*, we retrieved data from Oncomine and TCGA cohorts with analysis showing higher expression of *OPCML* and lower expressions of *LSAMP* and *NTM* in tumor tissues compared with normal tissue with statistical significance in LUAD (Figure 1B). In the Oncomine database, there were 6 (3 from LUAD, 2 LUSC and large cell carcinoma) and 1 LUAD cohorts showing downregulation of *LSAMP* and *NTM* genes, respectively. Based on these expression profiles, *LSAMP* was the most significant gene linked to lung cancer (Figure 1C). The available three cohorts focusing on LUAD patients were listed in Figure 1D. Taking these data into consideration, *LSAMP* and *NTM*, but not *OPCML*, in the IgLON family acts as a tumor suppressor in lung cancer. 

### 3.2. LSAMP Expression Is Negatively Correlated with Tumor Aggressiveness

**Figure 2:**
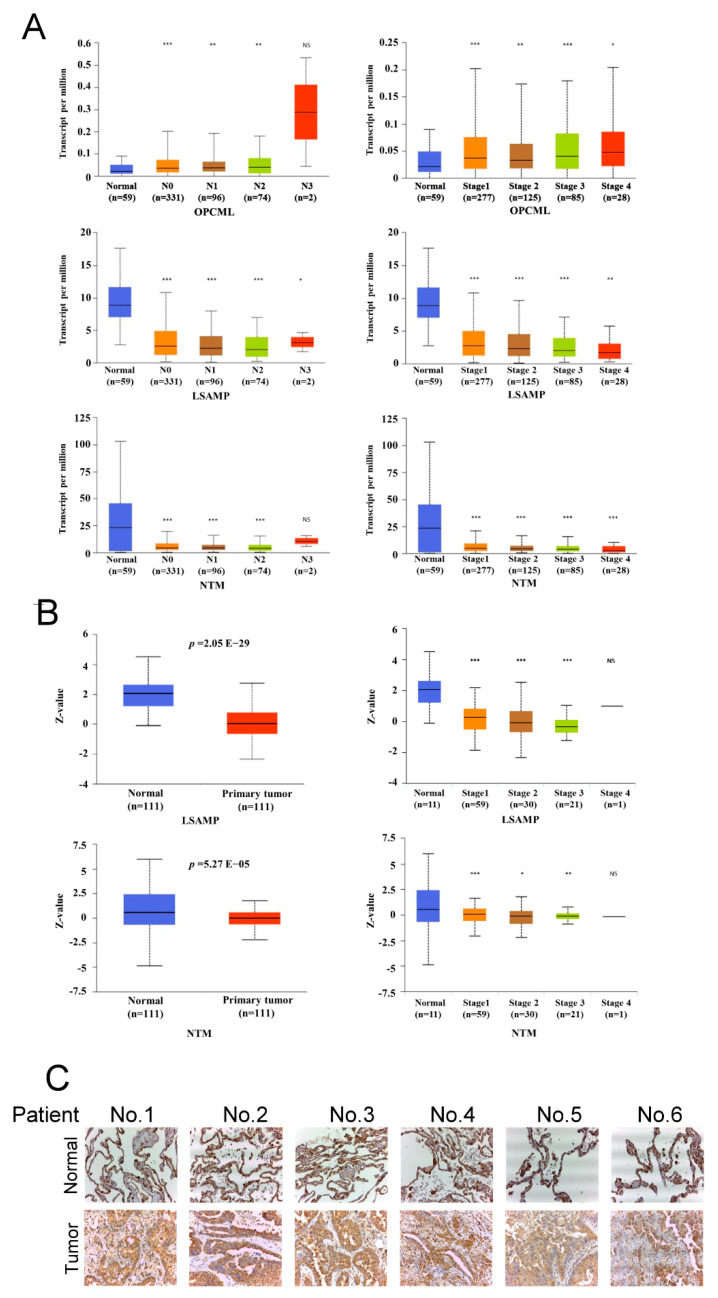
Low expression of LSAMP conferred lung tumor aggressiveness. The TCGA cohort showed the relationship between mRNA levels of *OPCML* (upper panel, (**A**)), *LSAMP* (middle panel, (**A**)), *NTM* (lower panel, (**A**)) and nodal metastasis and tumor stages. Furthermore, the CPTAC cohort showed the protein levels, LSAMP, and NTM (left panel, (**B**)) in both normal and tumor tissues. The correlation between the protein expression levels of LSAMP and NTM and tumor stages (right panel, **B**). The immunohistochemical staining of LSAMP from six lung cancer patients (**C**). * *p* < 0.05; ** *p* < 0.01; *** *p* < 0.005; ns, not significant.

**Figure 3:**
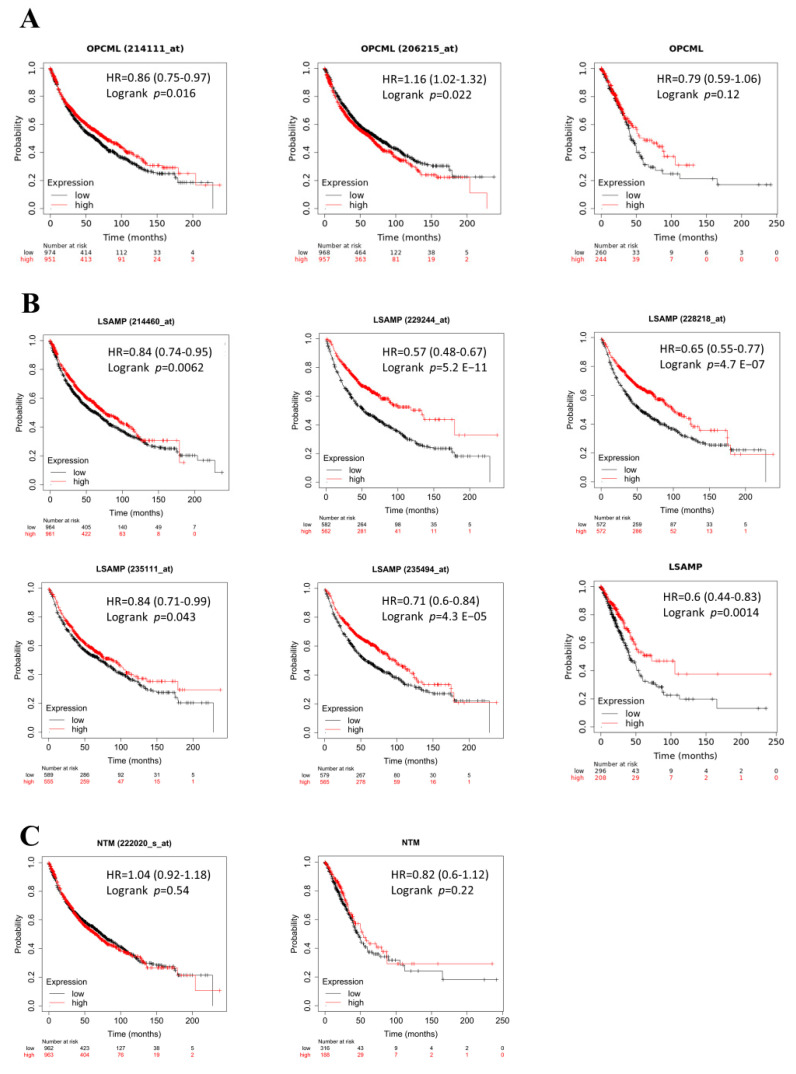
The survival analysis of IgLONs. Survival advantage or disadvantage should signify the role of each gene in lung cancer. Each gene of IgLONs and its K-M survival analyses were listed as *OPCML* (**A**), *LSAMP* (**B**), and *NTM* (**C**), respectively. HR, hazard ratio.

Concerning the function as suppressors of IgLONs in tumor aggressiveness, nodal metastasis and tumor staging were adopted for analysis. The expression of *OPCML* had no significant difference in nodal metastasis and tumor stages in the TCGA cohort (upper panel, Figure 2A). On the contrary, the expression of *LSAMP* and *NTM* correlated negatively with nodal metastasis and tumor stage (middle and lower panels, Figure 2A). The encoded genes are translated into specific proteins, which execute their specific physiologic function. Consistently, the protein expression of LSAMP and NTM of the CPTAC cohort revealed that tumor parts expressed lower levels of LSAMP and NTM protein in LUAD (left panel, Figure 2B); furthermore, the levels of LSAMP and NTM protein were correlated negatively with tumor stage (right panel, Figure 2B). However, the difference in LSAMP was more significant than that of NTM. LSAMP expression was lower in lung tumor parts than in lung normal parts from six lung cancer patients (Figure 3C). In summary, the expression of *LSAMP* was negatively correlated with tumor aggressiveness in both RNA and protein levels, which suggests that low expression of *LSAMP* is involved in tumor progression in LUAD.

### 3.3. The Lower Levels of LSAMP Conferred Poor Survival Prognosis Clinically

To further verify the prognostic value of IgLONs in LUAD, the survival analyses were retrieved from the K-M plotter. The expression of *OPCML* (Figure 3A) did not correlate with survival and neither did *NTM*, but the survival analyses of *LSAMP* illustrated survival disadvantage from low *LSAMP* expression in LUAD extracted from the KM plotter. (Figure 3A–C). The aggressiveness of cancer shortens survival time in cancer patients and based on the survival data, lower expression of *LSAMP* but not *NTM* conferred shorter survival time in lung cancer. This suggests that *LSAMP* exhibits survival benefits in LUAD patients.

### 3.4. The Epigenetic Regulatory Mechanisms for LSAMP Expression

**Figure 4:**
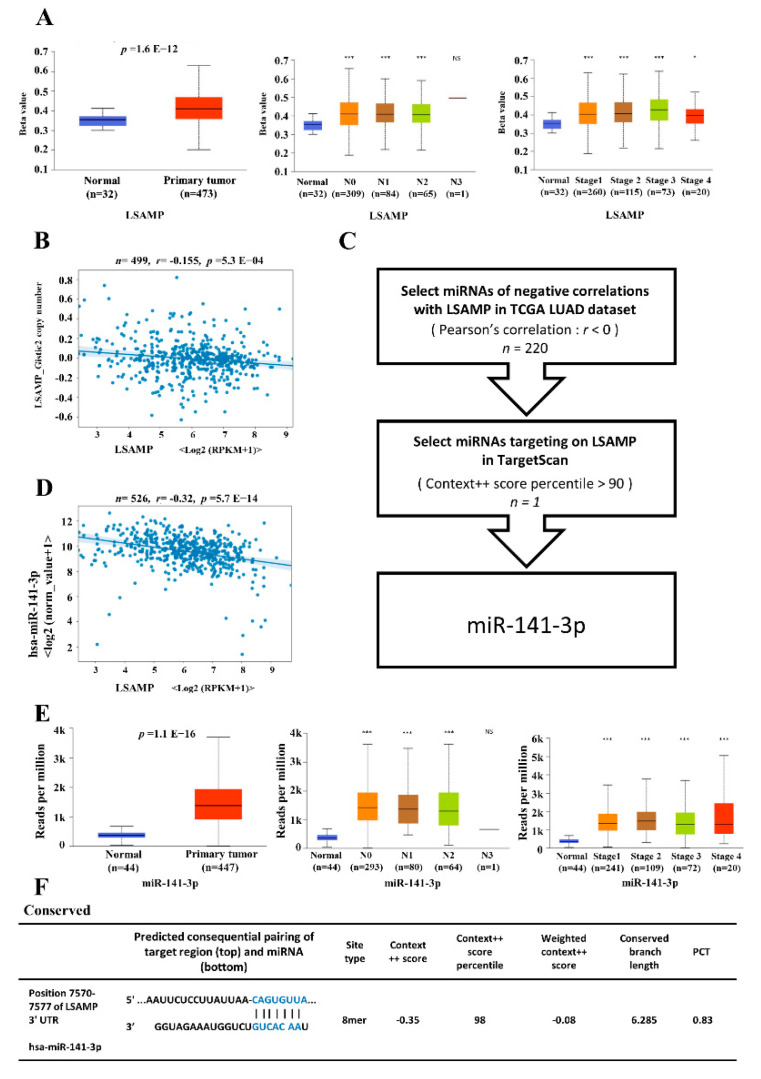
The regulatory mechanisms for *LSAMP* expression. The possible regulatory mechanisms included DNA methylation and nodal metastasis and tumor stage (**A**) and the correlation between copy number variation and *LSAMP* expression (**B**). The flow chart for the prediction of negative correlation between miRNAs and *LSAMP* from TCGA showed in Panel (**C**) with its r-value of 0.32 (**D**). The high binding score predicted from TargetScan was 98% (**E**). The correlation between miR-141-3p expression and tumor, nodal metastasis, and stage (**F**). * *p* < 0.05; *** *p* < 0.005; ns, not significant.

The expression of each gene is tightly controlled by epigenetic mechanisms as a DNA transcription step to posttranscriptional regulations. To elucidate the dysregulating mechanisms of *LSAMP*, possible regulatory mechanisms such as DNA methylation, copy number variation, and miRNAs were explored. Hypermethylation in the promotor of *LSAMP* was found in the tumor parts of LUAD, which correlated negatively with *LSAMP* mRNA. In addition, the status of hypermethylation of *LSAMP* correlated with nodal metastasis and higher tumor stages (Figure 4A). However, the correlation between copy number variation and expression of *LSAMP* was irrelevant (Figure 4B). To further elucidate the post-transcriptional control by microRNA, the TCGA cohort was utilized to find possible candidates, as validated by 3’UTR binding ability of microRNAs predicted by TargetScan. With the aid of bioinformatics, miR-141-3p was determined to be a regulator for *LSAMP* (Figure 4C). The binding prediction showed miR-141-3p has the ability to bind to 3’UTR of *LSAMP* from TargetScan (Figure 4E). The negative correlation between miR-141-3p and *LSAMP* was also proven from the TCGA cohort (Figure 4D). Moreover, the expression of miR-141-3p was higher in tumors compared with normal tissue parts, and its expression was higher in the advanced stage of LUAD, despite being not stage-dependent (Figure 4F). According to these data, both transcriptional and post-transcriptional modifications regulate *LSAMP* expression in LUAD.

### 3.5. The NEGR1 Interacts with LSAMP Expression

**Figure 5:**
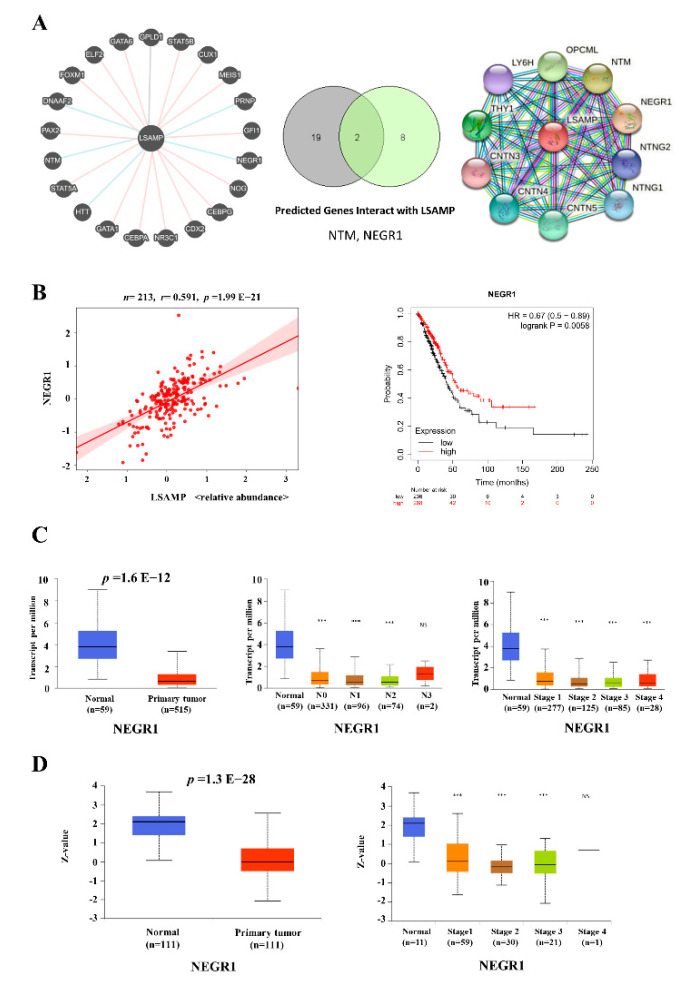
NEGR1 has an interaction with LSAMP. *NTM* and *NEGR1* were the predicted genes interacting with *LSAMP* by intersecting the results from websites, Pathway Commons (left plot) and STRING (right plot) (**A**). NEGR1 had a good correlation with LSAMP and positively linked to better survival (**B**). *NEGR1* mRNA expression was suppressed in LUAD, and associated with lymph node metastasis and advanced tumor stages (**C**). NEGR1 protein has lower expression in tumor parts in LUAD and correlates with advanced tumor stages (**D**). *** *p* < 0.005.

Signaling transduction pathways mediate the underlying mechanism of cell growth, cell apoptosis, organismal development, and pathways-aberrant diseases [29]. To elucidate the signaling transduction pathway, the Pathway Commons (gene, version 12) and STRING (protein, version 11.0b) were utilized to predict the protein interaction of LSAMP. There were two common genes/proteins, including NTM and NEGR1 predicted by the intersection of Pathway Commons and STRING (Figure 5A). In addition, a positive correlation (r = 0.591) between LSAMP and NEGR1 further confirmed the interaction between these two genes/proteins (left panel, Figure 5B). In addition, higher NEGR1 expression conferred longer survival time in LUAD patients (right panel, Figure 5B). Furthermore, the mRNA expression of *NEGR1* was lower in tumor parts compared with normal parts (left panel, Figure 5C), and its expression was correlated negatively in nodal metastasis and staging from TCGA (right two panels, Figure 5C). The protein expression of NEGR1 was lower in tumor parts compared with normal parts (left panel, Figure 5D) and its expression was correlated negatively in staging from the CPTAC cohort (right panel, Figure 5D). Based on these data, NEGR1 might interact with LSAMP, which affects the clinical outcomes of LUAD.

### 3.6. LSAMP Interferes with Epithelial-Mesenchymal Transition

**Figure 6:**
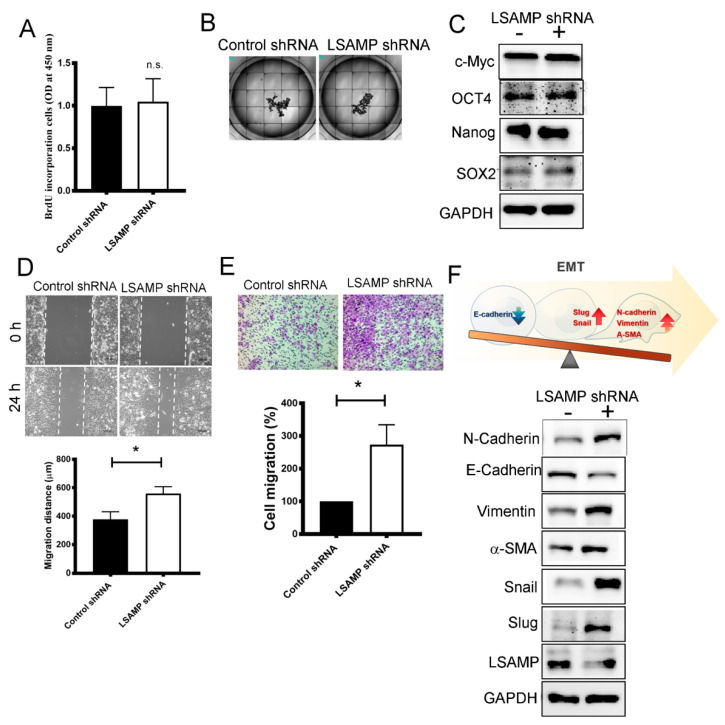
Loss of function in *LSAMP* enhances lung cancer progression. Cellular functions changed after knocking down *LSAMP*. Several cellular behaviors were investigated as proliferation, as determined by BrdU after 72 h incubation (**A**), tumor spheroid formation after 7-day culture (**B**), stemness (**C**), wound-healing assay (**D**), transwell migration after 48 h movement (**E**) and EMT markers and the illustration of EMT change. (**F**). All experiments were performed independently at least three times. The results were presented as mean ± SD. * Significant difference between the two test groups (*p* < 0.05).

To validate our previous data showing that *LSAMP* acts as a suppressor in tumorigenesis, functional assays were performed through an *LSAMP* shRNA knockdown model. With the aid of shRNA knockdown in a lung cancer cell line, A549, *LSAMP* did not affect cell proliferation through BrdU incorporation (Figure 6A), tumor spheroid formation (Figure 6B), nor stem cell characteristics (Figure 6C). On the contrary, cell migration, measured by both wound-healing and transwell migration assays, was enhanced after the loss of function in *LSAMP* (Figure 6D,E). The relevant mesenchymal markers, N-cadherin, vimentin, α-SMA, snail and slug strengthened after the knockdown of *LSAMP* via shRNA technique but an epithelial marker, E-cadherin, was attenuated (Figure 6F). These results reveal that the functional loss of *LSAMP* enables cancer cell migration via the trigging of EMT.

## 4. Discussion

Lung cancer has been one of the most deadly cancer for decades worldwide. However, the present treatment strategy is imperfect, especially in non-driver-mutation-type lung cancer [30]. In this study, advanced techniques, NGS and bioinformatics were utilized to discover and validate *LSAMP* in lung cancer tumorigenesis. Clinically, LUAD patients with low levels of *LSAMP* carried the worst clinical outcomes in several public datasets. Furthermore, the loss of function of *LSAMP* in lung cancer enhanced cellular migration via EMT under the epigenetic regulation of DNA hypermethylation and miR-141-3p. Our study indicated that *LSAMP* acts as a suppressor in lung cancer tumorigenesis. This study provides evidence that *LSAMP* would be a good candidate target for developing a treatment strategy in non-driver mutation lung cancer.

*LSAMP* has been known as a tumor suppressor gene in osteosarcoma and ovarian cancers [20,31]; however, the evidence of *LSAMP* functions for lung cancer tumorigenesis is still lacking. By utilizing the NGS data from six paired lung normal/tumor tissues and public datasets, this study found that tumor tissues expressed low-level *LSAMP* compared with normal tissues in LUAD. To verify its role in LUAD, several public datasets confirmed that low levels of *LSAMP* conferred tumor invasiveness, nodal metastasis, and advanced tumor stages as well as poor clinical outcomes with short survival time. To elucidate the role of the loss of function in *LSAMP* in tumorigenesis, several molecular mechanisms have been explored through shRNA knockdown. The wound-healing assay revealed enhanced wound closure as increased migration through EMT but not proliferation, tumor spheroid formation, or stemness in this study. LSMAP belongs to the superfamily of cell adhesion molecules (CAMs), which are the most abundantly expressed glycosylphosphatidylinositol (GPI)-anchored cell surface glycoprotein [32]. CAMs act as a subset of adhesion proteins on the cell surface and might affect cellular mechanisms of growth, contact inhibition, and apoptosis [33]. The specific characteristic of LSAMP as a CAM might explain its function in lung cancer progression.

To investigate the regulation over *LSAMP*, several proposed regulatory mechanisms as DNA methylation of the promoter region and copy number might regulate *LSAMP* expression [22,31]. In this study, the DNA methylation and the miR-141-3p negatively correlated with *LSAMP* expression, but failed to identify the correlation with copy number variation of *LSAMP* through the TCGA cohort. In addition, the protein-protein interaction would affect the expression of candidate protein. Through the Pathway Commons and STRING open website, strong interaction in LSAMP-NTM and LSAMP-NEGR1 was discovered. Reports illustrate the number of potential interactions between IgLONs due to their ability to form homophilic and heterophilic bonds, both in cis and trans orientations [32]. The interaction among IgLONs might be worthy of further investigation.

In this study, a series of investigations *in vitro* suggested that loss of function of *LSAMP* enhanced migratory ability via EMT but not proliferation or stemness in LUAD. However, this study has some limitations. Firstly, with only six paired samples, a definite conclusion for the role of *LSAMP* in lung cancer might not be reachable. This limitation might be improved by using public datasets. Secondly, the regulatory mechanisms for the downregulation of *LSAMP* were predicted via public databases. However, with this solid molecular evidence, this study provides the role of *LSAMP* in LUAD tumorigenesis and might offer hope for lung cancer patients.

## 5. Conclusions

Based on our study results, *LSAMP* acts as a suppressor in lung cancer tumorigenesis and *LSAMP* might be of great potential in developing a therapeutic strategy to overcome lung cancer.

## Figures and Tables

**Table 1:** Patient characteristics.

Number	Gender	Age	Stage	AJCC, 8th Ed.	Comorbidity
01	M	70	2B	T3N0M0	Old stroke
02	M	66	4B	T2aN0M1c	Hypertension
03	F	51	1B	T2aN0M0	Hepatitis B carrier
04	M	53	3A	T3N2M0	Hypertension
05	F	60	1A	T1bN0M0	Hepatitis B carrier
06	M	67	1A	T1aN0M0	UTUC, 2016

UTUC, Upper Tract Urothelial Carcinoma.

## Data Availability

All data are available upon request.
